# Motivations for inadequate persistence with disease modifying anti-rheumatic drugs in early rheumatoid arthritis: the patient’s perspective

**DOI:** 10.1186/1471-2474-14-336

**Published:** 2013-12-01

**Authors:** Virginia Pascual-Ramos, Irazú Contreras-Yáñez

**Affiliations:** 1 Department of Immunology and Rheumatology, Instituto Nacional de Ciencias Médicas y Nutrición Salvador Zubirán, Vasco de Quiroga 15, colonia sección XVI, Tlalpan, México DF 14000, México

## Abstract

**Background:**

Knowledge of factors that contribute to non-persistence with disease modifying anti-rheumatic drugs (NP) is essential to improve rheumatoid arthritis (RA) outcomes. Aims of the study were to investigate patient’s motivations and risk factors for NP in a cohort of early RA patients.

**Methods:**

Up to September 2012, data from 149 patients, who had at least 1 year of follow-up, at least one drug indication, and at least 2 consecutive six-months-apart rheumatic evaluations that included assessment of compliance were reviewed. NP and patient’s motivations of NP were evaluated according to a questionnaire. NP was defined when patients referred that they had completely stop RA medication, “Sometimes”, “Almost always” or “Always”. Patients had to pay for their medication.

Descriptive statistics and logistic regression models were used. Statistical significance was set at a p value of less than 0.05. The study was approved by the internal review board.

**Results:**

Up to cut-off, 715 questionnaires were applied to 149 patients, who had follow-up of 58.7 ± 27.9 months and were indicated 2.4 ± 0.7 DMARDs/patient/follow-up.

Patients were most frequently female (88.6%), middle-aged ([mean ± SD] age of 38.5 ± 12.8 years) with lower-middle/lower socio-economic status (87.9%) and scholarship of 11 ± 3.9 years.

Ninety-nine (66.4%) patients were NP and filled 330 questionnaires. Multivariate analysis showed that years of formal education (OR: 1.12, 95% CI: 1.1-1.24, p = 0.03), perception of at least some difficulty to find arthritis medication (OR: 5.68, 95% CI: 2.48-13, p = 0.000) and perception that arthritis medication is expensive (OR: 5.27, 95% CI: 2.1-13.84, p = 0.001) at the first evaluation of patient’s compliance were all predictors of NP.

Among the 99 NP patients, 25 (25.3%) were recurrent-NP and accumulated more disease activity. The combination of both reasons of NP (“Because it was not available at the drugstore” and “Because the medication is very expensive”) when selected at the first evaluation of compliance was the only variable to predict recurrent NP, OR: 4.8, 95% CI: 1.1-20.8, p = 0.04.

**Conclusions:**

Health systems should provide (first line) treatment for RA as a strategy to improve compliance with therapy and clinical outcomes, particularly in vulnerable populations.

## Background

Rheumatoid arthritis (RA) is a chronic inflammatory disease that may result in significant disability, morbidity and increased mortality [1-3]. Patients from Latin-America present unique and distinctive epidemiological, serological and clinical features regarding their disease. Published literature highlights a lower prevalence [4], a younger age at presentation [4,5] and a less severe clinical expression [5] when compared to Caucasians.

Current RA treatment guidelines recommend early aggressive management with disease modifying anti-rheumatic drugs (DMARDs) in order to improve patient’s outcomes. However, poor compliance with therapy is a substantial problem that affects 20% to 70% of the patients during follow-up [5-16]. Local experience with a cohort of Mexican Mestizo patients with early disease have confirmed these data and additionally shown that poor compliance with traditional DMARDs was associated with increased disease’s flares, decreased rates of remission and worse patient-reported outcomes [5,17,18]. The impact of inadequate therapy behavior in (early) RA patient’s outcomes may be further amplified by the fact that almost all the patients who had poor compliance with drugs eventually dropped out of treatment completely [19]. Furthermore, as a result of undetected or unreported therapeutic non-compliance, physicians may recommend a more complex regimen, which may increase costs and adverse events risks.

Many factors have been related to poor compliance with drugs in patients with RA [20] and include younger age [9,14], male sex [13,14], belonging to ethnic minority [19], lower education [10], side effects [10], availability of financial resources and social support [11], medication taking behavior and beliefs [12], increased disability [13], better perceived health status at the beginning [6], poor quality of contact with health professionals [13], poor personal knowledge about the disease and its treatment [13] and class of DMARDs [8,15]. Published data are obtained from studies performed in developed countries and in populations predominantly Caucasians.

In 2004, we establish an Early Arthritis Clinic in a referral Centre for Rheumatic Diseases in México City. Since the beginning, patient’s medication behavior has been assessed, initially through a structured interview and since November 2008 through an instrument locally designed, *the Compliance Questionnaire (CQ)*, that evaluates both constructs, adherence with and persistence (P) on therapy and investigates patient’s motivations of inadequate persistence with therapy [16].

In addition to the distinctive features above mentioned, RA patients from Latin-America are frequently uninsured, had a low socio-economical status and are less educated than RA patients from developed countries that may additionally impact compliance and outcomes. From the perspective of achieving desirable clinical outcomes, the negative effect of poor compliance with DMARDs needs to be minimized. However, in order to formulate effective strategies to contain the problem there is a need to investigate the factors that contribute to poor compliance. The aims of the study were:

1. To investigate most frequent patient’s motivations for non-persistence with disease modifying anti-rheumatic drugs (NP) in early RA Mexican Mestizo patients.

2. To identify risk factors of both, NP and recurrent NP.

## Methods

### Setting and study population

The early arthritis Clinic at the Instituto Nacional de Ciencias Médicas y Nutrición was initiated in 2004. Patients entering the Clinic had disease duration of less than a year when first evaluated and no specific rheumatic diagnosis but with RA. Patients are evaluated every two months during the first 2 years of follow-up and thereafter every 2, 4 or 6 (fixed for all the patients from the baseline evaluation) months depending on patients and disease characteristics. Treatment is prescribed by the rheumatologist in charge of the Clinic and is “treat to target” oriented.

Our Center belongs to the National Institutes of Health from México. Patients had health expenditures government coverage depending on their incomes; coverage varies from 0 to 100%. Most of the patients (70%) had 70% of their expenditures covered. Costs that are partially covered include: medical consultations, hospitalizations, emergency room and critical care unit, laboratory and all the diagnostic procedures available. Patients need to pay for their medication and these are not provided by the local pharmacy (unless patients are hospitalized and if available).

### Rheumatic evaluations

At study entry a complete medical history and demographic data are recorded along with disease specific autoantibodies. Medical evaluations are standardized and included swollen and tender joint counts, acute reactant-phase determinations, patient- and physician- reported outcomes, comorbidity, and treatment’s assessment (name/s, dose/s and schedule/s of all drug/s they were taking since last visit) along with the evaluation of compliance with therapy.

### Therapy behavior evaluation

Since 2008, the CQ is applied at fixed- six month intervals to all the patients. The *CQ* is a 22-items questionnaire (Additional file 1) which evaluates both adherence with and persistence on therapy. Briefly, Items 1 and 2 are related to demography; items 3 and 4 are related to the use of alternative medicine; items 5 and 6 evaluate patient-physician relationship; in item 7 patients qualify the quality of physician’s evaluation and central laboratory facilities; item 8 evaluates availability of medication at the drugstores and item 9 evaluates medication cost patient’s perception, both of them use Likert scales; in items 12 to 14, patients use a Likert scale to evaluate adherence to DMARD therapy; items 15, 17, 19 and 21 investigate patient’s knowledge about the disease; finally, items 16, 18, 20 and 22 determine the level of social support.

In item 10, patients use a Likert scale (0 to 4) to determine NP; patients who scored item 10 as 1, 2, 3 or 4 are directed to answer item 11 mean while those who score it as 0 are directed to proceed to item 12; item 11 investigates patient’s reasons of inadequate medication taking behavior and includes 15 predefined answers (most of them obtained from literature review) and one open answer.

Performance of the CQ has shown high sensitivity and satisfactory specificity to detect persistence on DMARDs [17]. CQ is fulfilled without help by 95% of the patients.

### Definitions

Disease activity was defined according to cut-offs of the Disease Activity Score, when 28 joints are examined (DAS28), [21,22].

Disability was evaluated according to the HAQ [23].

A patient was considered to be P*ersistent* if in item 10 “In the past 6 months, how often did you completely stop taking your medication? “ boxes 0 (“Never”) or 1 (“Almost never”) were filled.

A patient was considered to be *NP* if in tem 10 boxes 2 (“Sometimes”), 3 (“Almost always”) and 4 (“Always”) were filled.

A patient was considered to *be recurrent-NP* if he/she was defined as NP at every time the CQ was applied.

### Ethics

The study was approved by the internal review board (*Comité de Ética en Investigación del Instituto Nacional de Ciencias Médicas y Nutrición Salvador Zubirán*). Written informed consent was obtained in order to have patient’s charts reviewed and data presented in scientific forums or published.

### Statistics

Student t test, one-way ANOVA and X^2^ were used for normally distributed variables and Mann–Whitney U for non-normally distributed variables.

To identify baseline predictors for NP and recurrent-NP, logistic regression models were used. Those variables bivariately showing a significance level of p ≤ 0.05 were included into a regression model. Previously, correlation between selected variables was analyzed. The full multivariate model was reduced by stepwise removal of baseline variables with a significance level of p ≤ 0.05.

Statistical analysis was performed using the SPSS/PC program (v.12.0; Chicago IL). Statistical significance was set at a p value of less than 0.05.

From the present report, data from patients with at least 1 year of follow-up, at least one DMARD indication over follow-up, and at least 2 consecutive six-months-apart evaluations for compliance were reviewed. Up to September 2012, 149 patients met the inclusion criteria, which corresponded to 93% of the population of the Early Arthritis Clinic.

## Results

### Characteristics of the population

Up to cut-off, 715 CQ were applied to 149 early RA patients who had (mean ± SD) follow-up of 58.7 ± 27.9 months and were indicated (mean ± SD) 2.4 ± 0.7 number of DMARDs/patient/follow-up. Main characteristics of the patients at cohort inclusion are summarized in Table 1.

**Table 1:** Characteristics of the population at the baseline evaluation

**Variables**	**Population**
	**N = 149**
**Demographic**	
Females, N° (%)	132 (88.6%)
Age at baseline, years, mean ± SD	38.5 ± 12.8
**Years of education, mean ± SD**	11 ± 3.9
**Marital status of the patients, N° (% of the patients)**	
Married	72 (48.3)
Single	60 (40.3)
Separated/divorced/widow	17 (11.4)
**Occupation of the patients, N° (% )**	
Formal occupation	56 (37.6)
Housewife	54 (36.2)
Willing to work	20 (13.4)
Students	12 (8.1)
Informal occupation	7 (4.7)
**% of health expenditures with government coverage**	
N° (%) of patients with 90% coverage	3 (2)
N° (%) of patients with 80% coverage	25 (16.8)
N° (%) of patients with 70% coverage	104 (69.8)
N° (%) of patients with 60% coverage	17 (11.4)
**Disease specific autoantibodies**	
N° (%) of patients with Rheumatoid Factor	117 (78.5)
N° (%) of patients with antibodies to cyclic citrullinated proteins	117 (78.5)
**Disease characteristics, median (range)**	
Disease duration, months	5 (3.2-6.8)
Disease Activity Score, 28 joints evaluated	6 (5.1-7.1)
Health Assessment Questionnaire	1.5 (0.9-2.1)
**N∘ (%) patients with ≥ 1comorbidity**	88 (59.1)
**Median (range) of comorbidity/patient**	1 (0–1)

N° = Number.

SD = Standard deviation.

### Patient’s motivations of NP

Among the 715 CQ applied, 330 (46.2%) were classified as NP. Table 2 exhibits patient´s motivations and their frequencies.

**Table 2:** Patient’s motivations for non-persistence and comparison of patient’s motivations during remission and disease activity states

	**N° (%) of times a motivation is referred among 330 CQ**	**N° (%) of times a motivation is referred among 109 CQ applied in remission states**	**N° (%) of times a motivation is referred among 221 CQ applied in active disease**	**P**
**Patient’s motivations**				
Because I forget to take it	143 (43.3)	43 (39.4)	100 (45.2)	0.38
Because I had no money to buy it	112 (33.9)	41 (37.6)	71 (32.1)	0.39
Because it was not available at the drugstore	100 (30.3)	32 (29.4)	68 (30.8)	0.89
Because I did not buy it	98 (29.7)	30 (27.5)	68 (30.8)	0.63
Because I was not at home when I had to take my medication	91 (27.6)	22 (20.2)	69 (31.2)	*0.05*
Because the medication is very expensive	79 (23.9)	27 (24.8)	52 (23.5)	0.91
Because I had to do more things than I usually do through the day	60 (18.2)	14 (12.8)	46 (20.8)	0.10
Because I went out on a trip	40 (12.2)	9 (8.3)	31 (14)	0.18
Because timing/s when my medication is prescribed is different from mealtime/s	30 (9.1)	11 (10.1)	19 (8.6)	0.81
Because I am taking a lot of medication at this time	25 (7.6)	13 (11.9)	12 (5.4)	0.06
Because it may me feel worse when I take it	23 (7)	9 (8.2)	14 (6.3)	0.68
Because it does not make me feel better	19 (5.8)	9 (8.2)	10 (4.5)	0.26
Because nobody reminded me to take my medication	15 (4.6)	1 (0.9)	14 (6.3)	*0.05*
Because nothing happens if I do not take it	8 (2.4)	5 (4.6)	13 (5.9)	0.82
Because I did fewer things than I usually do through the day	5 (1.5)	2 (1.8)	3 (1.4)	0.88

CQ = Compliance Questionnaire.

Every evaluation of compliance was performed as part of a complete rheumatic assessment which included disease activity evaluation as per DAS28. Accordingly, patients were classified as in remission (DAS28 ≤ 2.6) in 109 out of 330 evaluations and with disease activity in the remaining 221 evaluations. There were subtle differences in patient’s motivations of NP between active patients and patients who achieved remission: formers referred most frequently “I was not at home when I had to take my medication” and “nobody reminded me to take my medication” than their counterparts, as shown in Table 2.

### Predictors of NP

Ninety-nine (66.4%) patients were classified as NP at some point during their follow-up and filled 330 CQs, meanwhile 50 were classified as persistent (33.6%) and filled 385 CQs. P and NP patients were compared. No differences were found between groups regarding age, percentage of females, residence, occupation, marital status, health coverage and baseline percentage of patients with RF, baseline -disease activity,- comorbidity and –treatment, and cumulative-comorbidity and -treatment (data not shown). Nonetheless, NP patients had more years of formal education, longer follow-up, had more antibodies to cyclic citrullinated peptides (ACCP) and higher cumulative disease activity and disability than their counterparts, Table 3. Also, they selected more frequently responses “Slightly, Moderately, Quite a bit or Extremely” to the question (Number 8) “*In the past six months, how much difficulty did you had to find your arthritis medication at the drugstore*?” and to the question (Number 9)” *In the past 6 months, how much expensive did you consider was you arthritis medication?”* (Table 3).

**Table 3:** Differences between non-persistent (NP) and persistent patients

	**NP patients**	**Persistent patients**	**p**
	**N = 99**	**N = 50**	
**(Mean ± SD) years of formal education**	11.4 ± 3.8	10 ± 3.9	0.03
**(Mean ± SD) months of follow-up**	62.7 ± 26.4	50.1 ± 29.5	0.009
**N° (%) of patients with ACCP when entering the Clinic**	83 (84)	34 (68)	0.04
**(Mean ± SD) cumulative DAS28**	3.1 ± 1.8	2.2 ± 1.1	0.001
**(Mean ± SD) cumulative HAQ**	0.4 ± 0.4	0.3 ± 0.3	0.03
**N° (%) of patients who selected responses “Slightly, Moderately, Quite a bit or Extremely” to the question (Number 8),**	57 (57)	20 (39.5)	0.000
***“In the past six months, how much difficulty did you had to find your arthritis medication at the drugstore?”***			
**N° (%) of patients who selected responses “Slightly, Moderately, Quite a bit or Extremely” to the question (Number 9),**	92 (93)	42 (80)	0.002
***In the past 6 months, how much expensive did you consider was you arthritis medication***			

NP = Non-persistent.

N° = Number.

ACCP = Antibodies to cyclic citrullinated peptides.

DAS28 = Disease activity score (28 joints evaluated).

HAQ = Health assessment questionnaire.

Multivariate analysis showed that years of formal education (OR: 1.12, 95% CI: 1.1-1.24, p = 0.03), perception of at least some difficulty to find arthritis medication (OR: 5.68, 95% CI: 2.48-13, p = 0.000) and perception that arthritis medication is expensive (OR: 5.27, 95% CI: 2.1-13.84, p = 0.001) at the first evaluation of patient’s therapy behavior were all predictors of NP (age and gender correction was done).

### Predictors of recurrent NP

As it has been demonstrated that persistence impacts outcomes, we ought to define if there was a particular number of times a patient had to be NP in order to have greater disease activity and disability. Recurrent-NP patients had greater disease activity and disability than their counterparts, (mean ± SD) cumulative DAS28: 3.7 ± 2 vs. 2.9 ± 1.7, p = 0.04 and cumulative HAQ: 0.3 ± 0.2 vs. 0.1 ± 0.1, p = 0.05.

Among the 99 NP patients, 25 (25.3%) were recurrent-NP and their characteristics were compared to their counterparts (74 patients); no differences were found between groups besides those related to disease activity and disability. The number of patients with DAS28 remission was lower among recurrent-NP patients (45 vs. 8, p = 0.002) as was the number of patients with disease activity improvement according to European League Against Rheumatism (EULAR) categories (11 vs. 62, p = 0.000).

Logistic regression models were constructed in order to investigate predictors on recurrent-NP; in all of them, any possible combination of the 15 patient’s motivations of NP when referred at first evaluation of compliance were included. Models yield similar results: the combination of both reasons of NP (“Because it was not available at the drugstore” and “Because the medication is very expensive”) when mentioned at the first time the CQ is applied was the only variable to predict recurrent NP, OR: 4.8, 95%CI: 1.1-20.8, p = 0.04. Results were similar after correction for disease duration, gender and age at disease onset.

## Discussion

From the perspective of healthcare providers, therapeutic compliance is a major clinical issue in RA patients as it impacts disease’s outcomes [5,17,18]. Inadequate medication adherence (which includes three major components: persistence, initiation adherence and execution adherence) causes an increased financial burden for society as it has been associated to excess emergency care visits and hospitalizations, higher treatment costs and loss of productivity. Furthermore, as a result of undetected or unreported therapeutic non-compliance, physicians may change the regimen, which may increase the cost or complexity of the treatment and eventually the incidence of adverse events. One logical target in trying to complete the riddle of therapeutic non-compliance in RA would be to identify most common associated factors from the patient’s perspective and to identify predictors as we did in the present study developed in a well characterized population of Mexican Mestizo early RA patients.

We found that 2 out of 3 patients were classified as NP. In the literature, different studies have targeted adherence to DMARDs [5,6,9,10,13,15,16,24] and shown that the extent to which patients adhere to DMARD therapy varies between underuse and overuse. Such variations may be explained by differences in sample’s size, methods capturing medication adherence, variable disease duration, follow-up, disease activity and therapeutic modalities, although studies confirm that adherence in RA is suboptimal. An important point to consider is that medication adherence is a dynamic feature, not stable over time. We found that 25% of our patients were consistently NP similar to have been reported in longitudinal studies performed in other RA populations [5,14,25].

Factors identified from studies and reviews with poor compliance may be grouped into several categories, and divided into 5 domains according to the World Health Organization: namely patient-centered factors, therapy-related factors, healthcare systems factors, social and economic factors, and disease factors [20,26]. In our study, NP patients were directed to select at least one factor from a list; factors were included in the list as all of them have been shown to impact compliance in different populations [17,20,26]. Most frequent patient’s motivations for NP were forgetfulness, lack of financial resources and lack of availability at the drug store. Forgetfulness is a widely reported factor that causes non-compliance with both, medication and clinical appointments in different populations, including Mexican patients with type2-diabetes [27,28]. Meal frequency has been shown to be an effective tool to remind the patient to take his medications in Japanese patients [29] and this strategy could be intensified in order to improve compliance. Interestingly, in our study “forgetfulness” was highly correlated to the motivation “Because timing/s when my medication is prescribed is different from mealtime/s “(Rho: 0.92, p = 0.001, data not shown). Also, written instructions are better than oral advice for reminding patients to take their medications and we recommend it implementation for every patient, and at every appointment.

Patient’s perception of at least “some difficulty to find arthritis medication” (previously reported as lack of availability at the drug store) and that “arthritis medication is expensive” (lack of financial resources) were among the most frequent motivations for NP; when both of them were selected at the first evaluation of patient’s compliance, they predicted NP (in addition to a higher education level) and also recurrent- NP. Cost is a crucial issue in patient’s compliance especially for patients with chronic diseases [28,30]. A number of studies have shown that patients who had no insurance cover or who had low incomes (as our population of patients who had to pay for their medication) are more likely to be non-compliant when compared to patients with health insurance or relatively high incomes [27,31-34]. In RA patients, inadequate or nonexistent reimbursement by health insurance plans has shown to negatively affect adherence to biologics [16]. Also, among the identified health care systems factors that contribute to poor compliance are lack of availability and accessibility to healthcare [28]; a significant percentage of our patients selected lack of availability of the medication which is related to the former. Finally, regarding education level, intuitively it may be expected that patients with higher educational level should have better knowledge about the disease and therapy benefits and accordingly better compliance. García-González et al. [10] found an association between lower education and lower adherence in 102 ethnically diverse patients from Texas, among whom 72 had RA. However, similar to our findings other researchers have shown that non-RA patients with lower educations levels have better compliance [35,36]. One possible explanation may be that patients with lower education level might have more trust in physician’s advice. DiMatteo proposed that even highly educated patients may not understand their conditions and the benefits of being compliant [37].

In our study, most frequent patient’s motivations for NP were “unintentional” motivations. As opposed to intentional motivations, they reflect a person’s ability and skill at medicine taking, including forgetting, poor manual dexterity, losing medicines or not being able to afford them. Meanwhile, intentional non-adherence is a behavior driven by the decision not to take medicines [11,25,38]; drivers of this decision have been suggested to be based on patient’s beliefs about its illness and its treatment, which can be further categorized as perceived benefits and perceived concerns [25]. Neame et al. reported that most people with RA had positive beliefs regarding the necessity of their medication but levels of concern were also high and were positively associated to poor compliance as 91% of non-adherent RA patients had at least one concern about potential adverse events [39]. Besides the valuable conceptual distinction between intentional and non-intentional motivations of non-adherence in RA patients, a practical approach to poor patient’s medication behavior will be to identify individual main drivers of poor compliance and tailor the content of the adherence-improving-intervention to the individual patient’s motivation of non-adherence [40]. This comprehensive strategy may be more effective that traditional adherence intervention programs in RA that have shown inconsistent and limited effects [41-43]; furthermore, it can be applied in our population as 79% of our patients selected non-intentional motivations and among them, 66% selected exclusively non-intentional motivations during their follow-up meanwhile 28% selected both, intentional and non-intentional motivations (data not shown). This finding is in agreement with the fact that there were no major differences between active and inactive patients with NP.

Some limitations of the study need to be addressed. We did not use a well-validated questionnaire scale to assess persistence; we applied a short-patient-oriented questionnaire, locally designed which has shown adequate internal consistency, high sensitivity and satisfactory specificity to detect persistence on DMARDs [17]. We did analyze neither the construct of adherence nor major factors associated. This study was done in an inception cohort of early RA patients, with particular socio-demographic characteristics, ethnicity, treatment and health system and our results may not be generalized to RA populations with different characteristics [4].

Compliance with medication is a dynamic process and fluctuates over time; with a more extended length of follow-up, patients formerly classified as persistent with therapy may become non-persistent. We limited the study of factors associated to medication persistence, to patient’s motivations; ultimately, it is the patient who decides whether or not to take his/her medication as prescribed, and non adherent patient’s opinion are essential in order to design effective interventions. Finally, we investigate a limited number of patient’s motivations for non-persistence with medication although they were selected based on the existing literature.

## Conclusions

Almost half of Mexican Mestizo patients with early RA and partial health coverage do not take their disease modifying anti-rheumatic drugs as directed. Most frequent patient’s motivations for non persistence were non-intentional motivations as forgetfulness, lack of financial resources and lack of availability at the drug store. When patients identified concomitantly “some difficulty to find arthritis medication and that arthritis medication is expensive” at their first evaluation of compliance with therapy they may be at risk of non-persistence during their follow-up and of deleterious outcomes. Health systems should provide (first line) treatment for RA as a strategy to improve compliance with therapy and clinical outcomes.

## Abbreviations

RA: Rheumatoid arthritis; DMARDs: Disease modifying drugs; CQ: Compliance questionnaire; P: Persistence; NP: Non-persistence with disease modifying anti-rheumatic drugs; DAS28: Disease Activity Score (28 joints evaluated); HAQ: Health assessment questionnaire; ACCP: Antibodies to cyclic citrullinated peptides.

## Competing interests

The authors declare that they have no competing interests.

## Authors’ contributions

VPR participated in the design and conception of the study, performed clinical evaluations and acquired data, drove statistical analysis and drafted the manuscript. ICY participated in the study design and conception, performed statistical analysis and helped to draft the manuscript. Both authors read and approved the final manuscript.

## Pre-publication history

The pre-publication history for this paper can be accessed here:

http://www.biomedcentral.com/1471-2474/14/336/prepub

## Supplementary Material

Additional file 1 Compliance Questionnaire. Click here for file
