# Supplementary material for: Motivations for inadequate persistence with disease modifying anti-rheumatic drugs in early rheumatoid arthritis: the patient’s perspective
Source: BMC Musculoskelet Disord. 2013 Dec 1;14:336. doi: 10.1186/1471-2474-14-336 (PMC4219583; doi:10.1186/1471-2474-14-336)
Supplement: Additional file 1 — Compliance Questionnaire. [file 1471-2474-14-336-S1.pdf]

Compliance Questionnaire

Dear patient:

Medical treatments that help to control symptoms from diseases like yours are frequently indicated for a long period of time. Sometimes, patients forget or stop taking their medications, or missed a medical appointment what may account in lesser therapy effectiveness than previously expected.

We are interested in knowing possible reasons which may help you to continue taking your medication as prescribed in order to improve your medical attention.

Your participation in this study is voluntary. You may stop participating whenever you decide and if so, it will not interfere with the existing medical attention at the Institution.

You are invited to collaborate by answering the following survey.

This interview refers to the arthritis-therapy taking behavior you had since last visit to the outpatient early arthritis Clinic.

Interview date: Day, Month, Year

Name: First Last name, Second Last name, Name(s)

Institution identification number:

1 .- Actual occupation

1	Housewife	4	Non-officially employed	6	Retired
2	Student	5	Unemployed	7	Other
3	Officially employed				

2.- Socioeconomic classification at the Institution

1	90% gratuity	3	70% gratuity	5	50% gratuity
2	80% gratuity	4	60% gratuity	6	40% gratuity

3.- Have you taken any alternative therapy, additionally to the treatment prescribed by the rheumatologist in charge of your care?

1 Yes 2 No If the answer is yes please specified which one

4.- During the past 6 months, did you stop taking the medication prescribed by your rheumatologist because of any reason including the choice of alternative medicine?

4. Always 3. Almost always 2. Sometimes 1. Almost never 0. Never

5.- Please rate in a scale from 0 to 10, how much you trust your rheumatologist.

0 indicates no trust at all and 10 indicates all the possible trust.

6.- Please rate in a scale from 0 to 10, how well you have understood treatment indications given by the rheumatologist in charge of your care.

0 indicates no understanding of medical indications regarding treatment and 10 indicates a perfect understanding.

7a.- Please rate in a scale from 0 to 10 the quality of the rheumatic evaluations you received.

0 indicates the poorest quality and number 10 the best quality.

7b.- Please rate in a scale from 0 to 10 the quality of central laboratory appointments you received.

0 indicates the poorest quality and number 10 the best quality (excellence).

8.- In the past 6 months, how much difficulty did you had to find your arthritis medication at the drugstore

0 Not at all 1 Slightly 2 Moderately 3 Quite a bit 4 Extremely

9.- In the past 6 months, how much expensive did you consider was you arthritis medication

0 Not at all 1 Slightly 2 Moderately 3 Quite a bit 4 Extremely

10.- In the past 6 months, how often did you completely stop taking your medication?

4. Always 3. Almost always. 2. Sometimes 1. Almost never 0. Never

**If you have answered numbers 4 (always), 3 (almost always), 2 (sometimes) or 1 (almost never), please answer the following question as well (question number 11).*

**If you have answered number 0 (never), please go to question number 12*

11.- Please read the following sentences and cross with an X each sentence you consider it was a reason to stop taking your medication during the past 6 months. *You may choose more than one answer*

11.1- Because I had no money to buy it	Yes	No
11.2- Because it was not available at the drugstore	Yes	No
11.3- Because it does not make me feel better	Yes	No
11.4- Because it may me feel worse when I take it	Yes	No
11.5- Because the medication is very expensive	Yes	No
11.6- Because I forget to take it	Yes	No
11.7- Because nothing happens if I do not take it	Yes	No
11.8- Because I am taking a lot of medication at this time	Yes	No
11.9- Because I had to do more things than I usually do through the day	Yes	No
11.10- Because I did fewer things than I usually do through the day	Yes	No
11.11- Because nobody reminded me to take my medication	Yes	No
11.12- Because timing/s when my medication is prescribed is different from mealtime/s		
11.13- Because I was not at home when I had to take my medication	Yes	No
11.14- Because I did not buy it	Yes	No
11.15- Because I went out on a trip	Yes	No

* If you wish to write some other reason/s, you may do it in the following space……………………………………………………………………………

12.- In the past 6 months, I took my medication exactly at the day/s indicated by my rheumatologist

4. Always 3. Almost always. 2. Sometimes 1. Almost never 0. Never

13.- In the past 6 months, I took my medication exactly at the day-times indicated by my rheumatologist

4. Always 3. Almost always. 2. Sometimes 1. Almost never 0. Never

14.- In the past 6 months, every time I took my medication, I took the precise amount of tablets indicated by my rheumatologist

4. Always 3. Almost always. 2. Sometimes 1. Almost never 0. Never

15.- You consider that Rheumatoid Arthritis is ….

a) A chronic disease b) A disease that will resolve c) I do not know

16.- Do you have any confident to talk with? Yes No

17.- Do you consider that Rheumatoid Arthritis is a curable disease?

Yes No I do not know

18.- If you have an economical urgency is there somebody who can help you? Yes No

19.- Do you consider that Rheumatoid arthritis is an inherited disease?

Yes No I don't know

20.- If you have doubts about your health, is there somebody trustworthy to talk with? Yes No

21.- Do you believe that someone who has rheumatoid arthritis should exercise? Yes No I don't know

22.- Do you have relatives to talk or spend time with them? Yes No
